# Persistent DNA damage-induced premature senescence alters the functional features of human bone marrow mesenchymal stem cells

**DOI:** 10.1111/jcmm.12387

**Published:** 2015-01-26

**Authors:** Valentina Minieri, Silvia Saviozzi, Giovanna Gambarotta, Marco Lo Iacono, Lisa Accomasso, Elisa Cibrario Rocchietti, Clara Gallina, Valentina Turinetto, Claudia Giachino

**Affiliations:** a Department of Clinical and Biological Sciences, University of TurinOrbassano, Turin, Italy; b Department of Oncology, University of TurinOrbassano, Turin, Italy

**Keywords:** actinomycin D, DNA damage, mesenchymal stem cell, senescence-associated secretory phenotype, stress-induced premature senescence

## Abstract

Human mesenchymal stem cells (hMSCs) are adult multipotent stem cells located in various tissues, including the bone marrow. In contrast to terminally differentiated somatic cells, adult stem cells must persist and function throughout life to ensure tissue homeostasis and repair. For this reason, they must be equipped with DNA damage responses able to maintain genomic integrity while ensuring their lifelong persistence. Evaluation of hMSC response to genotoxic insults is of great interest considering both their therapeutic potential and their physiological functions. This study aimed to investigate the response of human bone marrow MSCs to the genotoxic agent Actinomycin D (ActD), a well-known anti-tumour drug. We report that hMSCs react by undergoing premature senescence driven by a persistent DNA damage response activation, as hallmarked by inhibition of DNA synthesis, p21 and p16 protein expression, marked Senescent Associated β-galactosidase activity and enlarged γH2AX foci co-localizing with 53BP1 protein. Senescent hMSCs overexpress several senescence-associated secretory phenotype (SASP) genes and promote motility of lung tumour and osteosarcoma cell lines *in vitro*. Our findings disclose a multifaceted consequence of ActD treatment on hMSCs that on the one hand helps to preserve this stem cell pool and prevents damaged cells from undergoing neoplastic transformation, and on the other hand alters their functional effects on the surrounding tissue microenvironment in a way that might worsen their tumour-promoting behaviour.

## Introduction

Human bone marrow mesenchymal stem cells (hMSCs) are adult multipotent stem cells with the capacity of self-renewal and ability to differentiate into adipogenic, chondrogenic and osteogenic lineages 1. The endogenous role for hMSCs is maintenance of stem cell niches (classically the haematopoietic), and as such, hMSCs participate in organ homeostasis, wound healing and successful ageing 2. The potential of hMSCs to maintain multipotency and proliferate extensively *in vitro* also provides new avenues for cell-based therapy in the restoration of damaged or diseased tissue and, currently, the numbers of clinical trials that employ MSCs are increasing 3,4. In the light of their physiological functions and therapeutic potential, the evaluation of hMSC response to genotoxic insults is of great interest.

Most of the established anti-cancer therapies cause various levels of DNA damage; cells respond through the activation of the DNA damage response (DDR) pathways that regulate cell cycle arrest and DNA repair. Generally, if DNA repair is ineffective, cells either undergo apoptosis or become senescent 5,6. The choice between these three outcomes is crucial for adult stem cells as it can dramatically impact on their pool preservation and functional features.

Actinomycin D (ActD) is a well-known antibiotic with anti-tumour activity, widely used for treating both adult neoplasms (*i.e*. gestational trophoblastic disease) and several paediatric malignant tumours (*i.e*. Wilms’ tumour, retinoblastoma, rhabdomyosarcoma, Ewing's sarcoma). Its biological activity is related to the ability to bind to the DNA duplex with high affinity, thereby interfering with transcription and replication 7. ActD was shown to accumulate in nucleated cells, such as bone marrow and tumour cells, to substantially greater concentrations than in plasma at corresponding times 8. We previously showed that ActD produces unrepairable DNA damage in both peripheral blood mononuclear cells and T cells from normal individuals 9, inducing their death *via* an ATM- and p53-dependent apoptotic pathway 9,10, but no data regarding ActD effects on adult stem cells, including hMSCs, are yet available.

Notably, hMSCs showed to be highly resistant to apoptosis induced by different genotoxic insults 11–17, even though the precise resistance mechanisms are not completely understood. Recently it has been reported that another possible response of hMSCs to injury is stress-induced premature senescence (SIPS) 18–24. Even if SIPS represents an important tumour suppressive mechanism that irreversibly prevents damaged cells from undergoing neoplastic transformation, it is clearly a double-edged sword 25. Senescent cells, indeed, develop the so-called senescence-associated secretory phenotype (SASP) 26,27, which is an altered secretory activity, that may induce changes in the tissue microenvironment in ways that can promote both cancer and ageing phenotypes 28,29.

This study aimed at investigating the molecular, cellular and functional changes induced in bone marrow-derived hMSCs by ActD treatment. We report that hMSCs react to this genotoxic insult by undergoing SIPS rather than apoptosis; that SIPS is induced by a persistent DDR activation hallmarked by enlarged γH2AX foci co-localizing with 53BP1 protein; that SIPS results in an overexpression of several SASP genes; and finally, that SIPS increases the capacity of hMSCs to promote motility of lung tumour and osteosarcoma cell lines *in vitro*. The present results underline a multifaceted response of hMSCs to ActD that might alter their functional effects on the surrounding tissue microenvironment.

## Materials and methods

### Cell culture and drug treatment

U2OS and CALU-1 cell lines were purchased from ATCC. hMSCs were obtained from Lonza Group (Walkersville, MD, USA), routinely expanded seeding at a density of 3500 cell/cm^2^ and subcultured twice a week. Exponentially growing hMSCs were seeded at 7000 cell/cm^2^ 24 hrs before genotoxic treatment. To minimize the effects of replicative senescence, hMSCs at early passages (p4–p7) were used. Adherent cells were cultured in complete DMEM (1000 mg/l glucose; Sigma-Aldrich, St Luis, MO, USA) supplemented with 10% foetal bovine serum (FBS; Sigma-Aldrich), 2 mM L-Glutamine, 1% kanamycin, 1% sodium pyruvate, 1% non-essential amino acids, 0.1% β-mercaptoethanol (all from Gibco, Gaithersburg, MD, USA).

Peripheral blood from four healthy donors was collected after signed informed consent. Peripheral blood mononucleated cells were isolated and T-cell lines were generated as described 30 and used when >95% of T cells were in G0/G1. T cells were cultured in RPMI supplemented with 2 mM L-Glutamine, 1% kanamycin, 1% sodium pyruvate, 1% non-essential amino acids, 0.1% β-mercaptoethanol (all from Gibco), 5% human serum (BioWitthaker, Cambrex, Baltimore, MD, USA), 200 U/ml IL-2 (from the myeloma producing cell line IL2-t6, kindly provided by Dr A. Lanzavecchia, IRB, Bellinzona, Switzerland).

ActD (A9415; Sigma-Aldrich) was dissolved at 10 μg/μl in DMSO (D5879; Sigma-Aldrich) and used at the final concentration of 0.5 μg/ml (400 mM) in complete medium. Cells treated with DMSO, diluted 1:20,000, were used as control. After 3 hrs of incubation, medium supplemented with ActD/DMSO was removed and cells were washed three times with PBS before being cultured in fresh complete medium for different recovery times. During long recovery times, medium was changed every 2–3 days to ActD-treated cells, whereas control cells were reseeded at a density of 3500 cells/cm^2^ to avoid overconfluence.

### Cell viability assay

Cell viability was evaluated by propidium iodide (PI) staining at 24, 48 and 72 hrs of recovery after cell treatment. Briefly, cells were collected, resuspended in PBS at 10^6^cells/ml and stained with 1 μg/ml PI for 5 min. at RT in the dark. At least 30,000 cells for each experimental point were acquired on a Cyan ADP flow cytometer (Beckman Coulter, Brea, CA, USA) and analysed with Summit 4.0 software. Viable cells were expressed as percentage of living cells, evaluated on PE-log *versus* FS-Lin plots, normalized respect to the relative time-point control.

### EdU staining

Human mesenchymal stem cells were seeded on μ-Slide 8 well ibitreat (Ibidi, Martinsried, Munich, Germany), treated with 2.5 μg/ml 5-ethynyl-2′-deoxyuridine (EdU) for 3 hrs and stained with the Click-iT® EdU Imaging kit (Molecular Probes Inc., Eugene, OR, USA), following the manufacturer's instructions. Briefly, hMSCs were fixed with 3.7% formaldehyde in PBS, permeabilized with 0.5% Triton X-100 and stained for 30 min. at RT in the dark with the Click-iT® reaction cocktail. hMSCs were then washed with PBS and stained with 5 μg/ml Hoechst 33342, for 20 min. in the dark, washed twice in PBS, mounted with Mowiol solution (Calbiochem, San Diego, CA, USA) and then analysed with 510 Carl Zeiss confocal laser microscope by using a 10× objective.

### Multipotent differentiation

For induction of either osteogenic or adipogenic differentiation, hMSCs were seeded at 30,000 cells/cm^2^. After 24 hrs of recovery from drug treatment, they were incubated in a differentiation medium for several weeks, with the medium being changed every 2–3 days. The osteogenic differentiation medium was as follows: DMEM 1000 mg/l glucose supplemented with 10% heat-inactivated FBS, 1% non-essential amino acids, 1% glutamine, 1% kanamycin (all from Gibco), 100 nM dexamethasone, 0.2 mM ascorbic acid 2-phosphate and 10 mM β-glycerophosphate (all from Sigma-Aldrich).

To detect mineralization (calcium deposits), 3 weeks after induction cells were fixed with ice-cold 70% ethanol and stained with 40 mM Alizarin Red S (Sigma-Aldrich). Images were taken with Motic AE2000 phase contrast microscope at 10× magnification. For adipogenic differentiation, the complete DMEM medium was supplemented with 10 μg/ml insulin, 1 μM dexamethasone, 0.5 mM 3-isobutyl-1-methylxanthine and 100 μM indomethacin (all from Sigma-Aldrich). To detect fat deposition, 2 weeks after induction cells were fixed with 10% formaline buffered solution for 1 hr at RT and stained with Oil Red O (Sigma-Aldrich). Images were taken with Motic AE2000 phase contrast microscope at 20× magnification.

### Senescence-associated β-galactosidase (SA-β-Gal) assay

Cell staining for β-galactosidase activity was performed as previously described by Debacq-Chainiaux *et al*. 31. The percentage of SA-β-Gal-positive cells was calculated by counting at least 1000 cells.

### Protein extracts and immunoblotting

Human mesenchymal stem cells were collected, washed with PBS, pelleted and lysed in Laemmli buffer (125 mM Tris-HCl pH 6.8, 5% SDS). Samples were boiled for 2 min. and, after chilling, Complete Mini protease inhibitor cocktail and PhosSTOP phosphatase inhibitor cocktail (Roche, Indianapolis, IN, USA) were added. Samples were then sonicated and centrifuged at 14,000 r.p.m. for 10 min. Protein content was determined by the micro-bicinchoninic acid method (Thermo Scientific, Rockford, IL, USA) and 20 μg of each total cell lysate were size fractionated by SDS-PAGE 3–8% gels (Invitrogen, Carlsbad, CA, USA) and electroblotted onto PVDF membranes (Amersham, GE Healthcare, Buckinghamshire, UK). After blocking with either 5% (wt/vol) non-fat dried milk in PBS plus 0.1% (vol/vol) Tween-20 (Sigma-Aldrich-Co.) or 5% (wt/vol) bovine serum albumin (BSA) in Tris-buffered saline, pH 7.5, the membranes were incubated with the following primary antibodies: mouse monoclonal antibodies against p53 1:1000 (clone DO7, Santa Cruz), phospho-p53(Ser15) 1:1000 (Cell Segnaling), β-actin 1:4000 (clone AC-74, Sigma-Aldrich), vinculin 1:4000 (clone HVIN-1, Sigma-Aldrich); rabbit monoclonal antibody against p16INK4A 1:1000 (clone EPR1473; Abcam, Cambridge, UK) and rabbit polyclonal antibody against p21 1:1000 (ab7960; Abcam). The secondary antibodies used include horseradish-conjugated goat anti-rabbit (Abcam) and horseradish-conjugated rabbit anti mouse (Abcam) (both 1:10,000). Immunoreactive bands were visualized by ECL Super Signal (Thermo Scientific) on autoradiographic films.

### Immunofluorescence

Human mesenchymal stem cells were plated on μ-Slide 8 well ibitreat (Ibidi, Martinsried, Germany), fixed with 4% paraformaldehyde, permeabilized with 1% Triton X-100 and blocked with 6% (wt/vol) BSA (Sigma-Aldrich) and 2.5% (vol/vol) normal goat serum (Sigma-Aldrich). Cells were then stained with the following primary antibodies: mouse monoclonal antibodies anti-phospho-Histone H2AX(Ser139) 1:500 (clone JBW301; Millipore, Billerica, MA, USA) and anti-ATMpS1981 1:300 (clone 10H11.E12, Rockland) or rabbit polyclonal antibody against 53BP1 1:500 (Novus Biological, Cambridge, UK) for 2 hrs at 4°C. Secondary antibodies were: Alexa 488 or Alexa 546 conjugated goat anti mouse and Alexa 546 conjugated goat anti-rabbit (all 1:500; Molecular Probes Inc.). Nuclei were stained with 0.1 μg/ml 4′-6-diamidino-2-phenylindole (DAPI; Sigma-Aldrich) and slides mounted by using Mowiol solution (Calbiochem). Fluorescence images were obtained with a TCS-SPE, Leica Microsystem at 63× magnification. γH2AX foci quantification was performed counting at least 300 cells for each time-point.

### RNA extraction, cDNA synthesis and real-time PCR

Human mesenchymal stem cells were dissolved in TRIzol reagent (Life Technologies) and total RNA (totRNA) was extracted according to the manufacturer's instructions. Genomic DNA contaminations were removed by DnaseI treatment (Ambion, USA) and RNA was then quantified. One microgram of totRNA was finally retrotranscribed with random hexamer primers and Multiscribe Reverse Transcriptase contained in High Capacity Reverse Transcription Kit (Applied Biosystems, USA) in accordance with manufacturer's suggestions. Expression levels of nine SASP genes (GM-CSF, GRO1, ICAM1, IL-6, IL-8, MCP-2, MMP3, RANTES and SDF1) were evaluated with SYBR green technology on an ABI PRISM 7500 Fast Real-Time PCR system (Applied Biosystems) by using 25 ng of cDNA template and 150 μM of each primer. Melting curve analysis was routinely performed to check for the presence of a single peak corresponding to the required amplicon. For each target gene, relative variation of transcript levels in ActD-treated hMSCs was evaluated with the ΔΔCt method by using Actβ as reference gene and time-matched DMSO-treated cells as calibrators. The primers used are listed in Table S1.

### Generation of conditioned medium, migration assay and proliferation assay

To generate hMSC-conditioned medium (hMSC-CM) from both control (DMSO-treated) and senescent (ActD-treated) cells, hMSCs were cultured for 24 hrs in DMEM supplemented with either 2% FBS for the migration assay or 10% FBS for the proliferation assay; then the CM was collected, spun down to remove cell debris (3000RPM for 10 min.) and passed through a 0.22 μm filter. CM aliquots were flash frozen in liquid nitrogen and stored at −80°C until required. For the migration assay, the lower chambers of 24-well systems were filled with 700 μl hMSC-CM; 100,000 CALU-1 or U2OS cells resuspended in 200 μl of 2% FBS DMEM were seeded in the upper part of the cell culture insert on a porous transparent polyethylene terephthalate membrane (8 μm pore size, 10^5^ pores/cm^2^; #353097; Becton Dickinson Biosciences). The multiwells containing the cell culture inserts were incubated at 37°C in a humidified 5% CO_2_ atmosphere; migration assay was carried out for 4.5 hrs (CALU-1) and 16 hrs (U2OS). Afterwards, culture inserts were rinsed with PBS and cells attached to the upper side of the membrane were mechanically removed by using a cotton-tipped applicator. Cells that migrated to the lower side of the membrane were fixed with 2% glutaraldehyde for 20 min., washed five times in water, stained 20 min. in 0.1% cristal violet, 20% methanol, washed five times in water and air-dried. For each cell insert, three images at 2.5× magnification were taken with a Leica DC100 phase contrast microscope and counted by using ImageJ software (Rasband, W.S., ImageJ, U. S. National Institutes of Health, Bethesda, MD, http://rsb.info.nih.gov/ij/, 1997–2013). The migration assay was performed in three independent experiments. For the proliferation assay, U2OS and CALU-1 cells were plated in triplicate at a density of 15,000 cells per well in 24-well plates. After overnight seeding, 500 μl of CM (either control or senescent hMSCs-CM) were added, tumour cells cultured for 48 hrs and their proliferation assessed by using the CellTiter-Blue Cell Viability Assay (Promega, USA). Fluorescence emission was read at 590 nm with a multi-plate reader (Infinite F200, Tecan, Switzerland).

### Statistical analysis

Data are represented as mean ± SD. For statistical analysis Student's *t*-test was performed and the significance was expressed with asterisks: *P < 0.05; **P < 0.01; ***P < 0.005.

## Results

### hMSCs are resistant to ActD-induced cell death

The survival of multipotent hMSCs following 3 hrs of ActD (400 mM) treatment was evaluated respect to two sensitive cell lines, differentiated-resting T cells 9 and the neoplastic-cycling osteosarcoma U2OS cell line 32,33.

**Fig 1:**
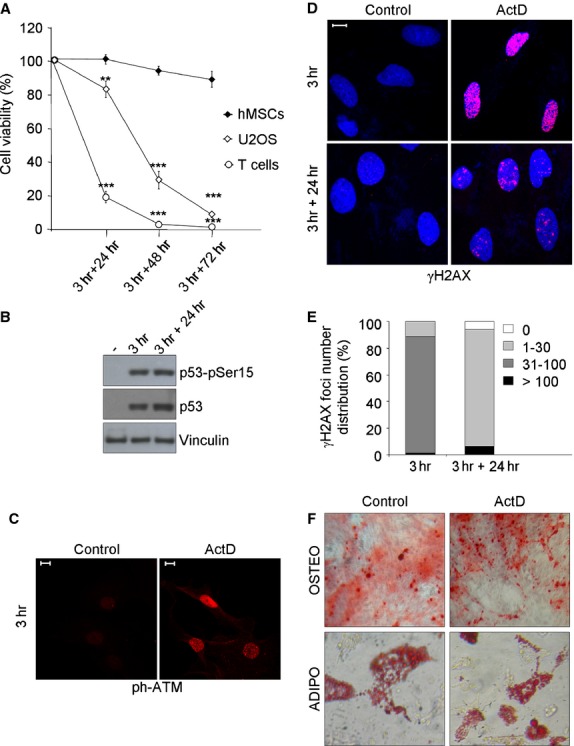
hMSCs are resistant to ActD-induced DNA damage. (A) Survival of ActD-treated cells was analysed by PI staining and flow cytometry. Cells were treated for 3 hrs with ActD (400 mM) and allowed to recovery in drug-free medium for 24, 48 and 72 hrs. Values were normalized on control cells and represented as mean ± SD of three independent experiments. Student's *T*-test was performed and the significance values indicated by asterisks. (B) Immunoblotting of total and phosphoSer15-p53 in hMSCs after 3 hrs of ActD treatment and after 24 hrs of recovery. Vinculin was used as loading control; sign (-) indicates control cells. (C) Immunofluorescence of ATM autophosphorylation in Ser1981 (ph-ATM) in hMSCs after 3 hrs of ActD treatment. (D) Immunofluorescence of γH2AX (red) foci induction in hMSCs after 3 hrs of ActD treatment and of residual foci after 24 hrs of recovery. DAPI counterstains the nuclei (blue). (E) γH2AX foci number quantification in ActD-treated hMSCs; one representative experiment out of two is shown. (F) Control and ActD-treated hMSCs were induced to differentiate after 24 hrs of recovery. Top panel: Alizarin Red S staining for calcium deposition after 21 days’ culture. 10× magnification images were taken with a contrast phase microscope. Bottom panel: Oil Red staining for lipid accumulation after 15 days’ culture. 20× magnification images were taken with a contrast phase microscope. In all panels the white bar represents 10 μm.

ActD induced an evident and significant cell death of both sensitive lines, with survival rates of 2.7% ± 1.5% (T cells) and 10.1% ±2.0 (U2OS) at 72 hrs. In contrast, hMSCs were resistant to ActD treatment with a survival rate of 87.6% ± 5.2% at 72 hrs (Fig.1A).

ActD induced a DDR response in hMSCs as demonstrated by autophosphorylation of ATM in Ser1981 and phosphorylation of p53 in Ser15 detected by immunofluorescence and western blotting respectively (Fig.1B and C). The phosphorylation of the histone variant H2AX on Ser139 (γH2AX), an early and specific marker of DNA damage, was then assessed. Immunofluorescence analysis showed that ActD treatment strongly induced γH2AX foci in hMSCs with almost 90% of cells carrying a number of foci comprised between 31 and 100 at 3 hrs of treatment (Fig.1D and E). After 24 hrs of recovery, only 6% of hMSCs had become completely negative for γH2AX, while the majority of cells still carried a number of foci comprised between 1 and 30 (Fig.1D and E). At this time-point a small fraction of cells (less than 7%) had more than 100 foci (Fig.1E), an event that could be ascribed to a small degree of apoptotic DNA fragmentation 34; accordingly, at 24 hrs of recovery about 8% of treated cells were found to be annexin V-positive/PI-negative, *i.e*. early apoptotic (data not shown).

Despite the presence of unresolved γH2AX foci at 24 hrs of recovery, hMSCs are still able to differentiate towards the osteogenic and adipogenic lineages. Indeed, after incubation with the respective induction media, both control and ActD-treated hMSCs showed the typical signs of osteogenic and adipogenic differentiation: extracellular calcium deposits that stained positive by alizarin red (Fig.1F, to panel) and intracellular lipid droplets that stain positive by oil red (Fig1F, bottom panel).

Taken together, these data demonstrate that hMSCs survive to ActD-induced DNA damage, respond with a quick and strong activation of the DDR pathway and retain their multipotent features, yet they maintain a considerable number of unresolved γH2AX foci.

### ActD-treated hMSCs undergo SIPS following a persistent DDR activation

Considering that DNA damage accumulation could result in SIPS 35, analyses were carried on to investigate if ActD-treated hMSCs underwent senescence.

**Fig 2:**
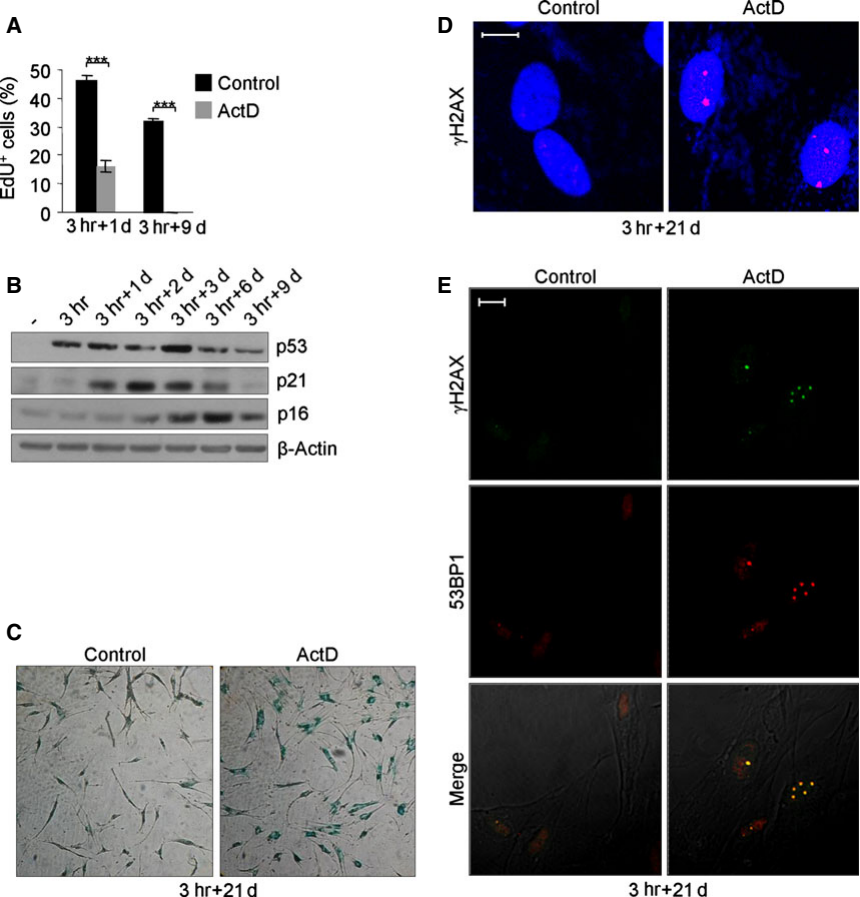
hMSCs activate SIPS following ActD treatment. (A) Percentage of EdU positive cells on the total Hoechst 33342 positive cells at 1 and 9 days of recovery after 3 hrs of ActD treatment. Mean ± SEM of three independent experiments is shown. Student's *t*-test was performed and the significance value indicated by asterisks. (B) Immunoblotting of p53, p21 and p16 proteins at 0, 1, 2, 3, 6 and 9 days of recovery. β-Actin was used as loading control. Sign (-) indicates control cells. (C) Representative images of β-galactosidase staining after 21 days of recovery (phase contrast microscope 10× magnification images). (D) Immunofluorescence analysis of persistent γH2AX foci (red) after 21 days of recovery. DAPI counterstains the nuclei (blue). (E) Immunofluorescence co-localization analysis of γH2AX (green) and 53BP1 (red) after 21 days of recovery. In all panels the white bar represents 10 μm.

As growth arrest is an hallmark of senescence, the inhibition of DNA synthesis was evaluated through EdU incorporation staining. After ActD treatment, a strong reduction of hMSCs in active DNA synthesis was observed already on day 1, with only 16% EdU positive cells, compared to 46% of EdU positivity detected in control cells. DNA synthesis was then completely abolished in ActD-treated cells by day 9 (Fig.2A).

Next, protein expression changes compatible with senescence induction were studied by immunoblotting during a 9-day interval (Fig.2B). p53 amount markedly increased soon after ActD treatment, it reached a maximum on day 3 and then started to decrease; after 9 days, the amount of p53 in ActD-treated cells was still slightly greater than in control cells (Fig.2B). Expression of the p53 responsive protein p21 protein, an inhibitor of the cyclin-dependent kinase 1A, was clearly detectable on day 1, reached its maximum on day 2 and subsequently gradually decreased (Fig.2B). Contemporarily to p21 decrease, the amount of p16, an inhibitor of cyclin-dependent kinases 4 and 6, increased and reached its maximum on day 6 (Fig.2B).

To further confirm senescence induction in ActD-treated hMSCs, the activity of SA-β-gal was evaluated. On day 3, the majority of ActD-treated cells were larger and more flattened compared with control hMSCs, which maintained a spindle-shaped, fibroblast-like morphology (data not shown). After 9 days, ActD-treated cells showed a marked SA-β-gal activity (55.3% ± 6.3%) compared with the controls (5.8% ± 2.3%; data not shown). The percentage of β-gal stained cells increased with time and reached its maximum after 21 days (84.7% ± 4.7% positive cells in ActD-treated cells *versus* 18% ± 1.2% positive cells in the controls; Fig.2C).

It has been reported that damaged senescent cells harbour characteristic enlarged and persistent DNA damage nuclear foci (PDDF) that contain DDR proteins, including γH2AX and 53BP1 36,37. In accordance, the presence of PDDF in ActD-treated hMSCs was observed: after 21 days of recovery about 50% of cells contained a number of γH2AX foci comprised between 1 and 10 (Fig.2D), that completely co-localized with 53BP1 foci (Fig.2E). These results strongly indicate that ActD treatment leads to a persistent DDR activation that causes SIPS in hMSCs.

### Senescent hMSCs display a SASP phenotype and promote tumour cell migration

**Fig 3:**
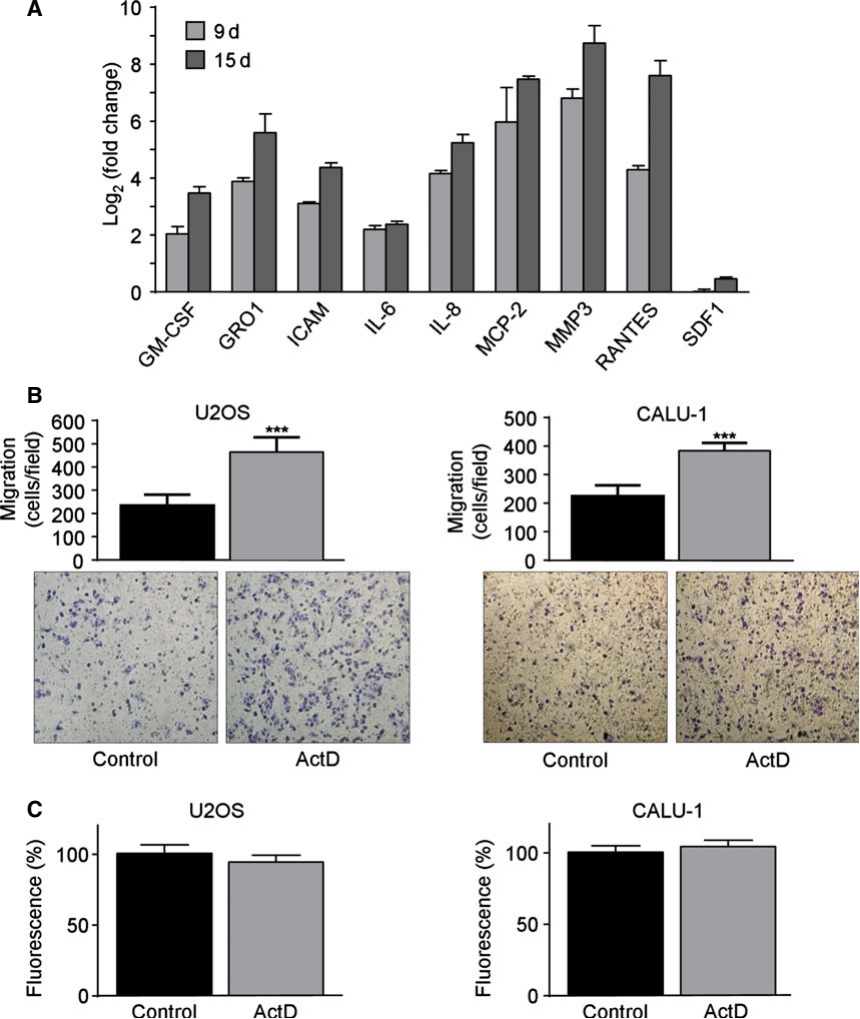
Senescent hMSCs augment the expression of inflammatory cytokine genes and promote tumour cell migration. (A) qPCR relative gene expression levels of nine SASP factors in ActD-treated hMSCs after 9 and 15 days of recovery. Relative variation of transcript levels in ActD-treated hMSCs was expressed as Log_2_ (fold change), calculated by using Actβ as reference gene and time-matched control cells as calibrator. One representative experiment out of two. (B) Influence of conditioned medium obtained from senescent and control hMSCs on the migration ability of U2OS and CALU-1 cell lines. Shown are representative images (bottom) and graph (top) of migrating cell number/field (mean ± SD) derived from three independent migration assays. Student's *t*-test was performed and the significance values indicated by asterisks. (C) Influence of conditioned medium obtained from senescent and control hMSCs on the proliferation of U2OS and CALU-1 cell line. CellTiter-Blue fluorescence values were normalized on control cells and represented as mean ± SD of three independent experiments.

Senescent cells increase the expression and secretion of numerous cytokines, chemokines matrix metalloproteinases and other proteins, the so-called SASP (reviewed 38). Considering that gene expression profiles of senescent cells determined by transcript analysis resemble the profiles of secreted proteins 26,39, the expression of nine SASP genes (GM-CSF, GRO1, ICAM1, IL-6, IL-8, MCP-2, MMP3, RANTES and SDF1) was analysed in senescent hMSCs after 9 and 15 days from ActD treatment. All genes, with the exception of SDF1, greatly increased their expression compared to control cells (Log_2_(fold change) ranging from 2.37 to 8.74) (Fig.3A).

Subsequently, the influence of hMSC-conditioned medium (hMSC-CM) of senescent and control cells on the motility and proliferation of two solid tumour-derived cell lines was investigated. Conditioned medium of senescent hMSC enhanced the cell migration ability of U2OS and CALU-1 by 2.0 and 1.7-folds, respectively, compared with conditioned medium of control hMSC (Fig.3B). On the other hand, CM of senescent hMSCs did not significantly influence proliferation of these two cell lines (Fig.3C).

Overall, these data suggest that senescent hMSCs alter their secretory profile, up-regulating the transcript expression of the two major inflammatory cytokines involved in SASP and enhancing the motility of tumour cells.

## Discussion

We investigated the molecular, cellular and functional changes induced in bone marrow-derived hMSCs by the anti-tumour agent ActD. We show that hMSCs activate the molecular pathway and exhibit the cellular features of SIPS and that SIPS is induced by persistent DDR signalling. Functionally, senescent hMSCs overexpress several genes involved in SASP and produce soluble factors able to promote tumour cell motility *in vitro*. Our findings disclose a multifaceted consequence of this chemotherapeutic treatment on hMSCs that on the one hand helps to preserve this stem cell pool and prevents damaged cells from undergoing neoplastic transformation, yet at the risk of altering hMSC effects on the surrounding tissue microenvironment.

While ActD had been previously shown to induce unrepairable DNA damage and to promote prompt apoptosis in cells of haematopoietic origin 9, here we found that ActD-treated hMSCs resist apoptosis and preferentially undergo senescence. Indeed, among the three established outcomes of the DDR activation (transient cell cycle arrest coupled with DNA repair, senescence and apoptosis), the first two appear to be preferable for an adult stem cell allowing at least partial preservation of the stem cell pool. Along this line, several *in vivo* and *in vitro* data have shown that hMSCs are particularly resistant to the apoptosis induced by DNA damage 11,14,15. This resistance to cell death relies on different mechanisms depending on the different DNA-damaging agents/doses used, including an elevated apoptosis threshold 16, an efficient antioxidant ROS-scavenging capacity 13 and a prompt activation of the double strand break repair pathways 13,17. Our results are in accordance with the more recent literature that described SIPS as a further response of hMSCs to ionizing radiation 21,40, oxidative stress 18,41, heat shock 19.

One of the main characteristics of SIPS is the accumulation of persistent γH2AX foci, differing, both in size and persistence, from the transient foci that occur during initial successful DSB rejoining. These PDDF have been described in many senescent differentiated cell types both *in vitro* and *in vivo* 36,37,42. Here we describe the presence of PDDF in undifferentiated stem cells and, in accordance with the observations made in differentiated cells, persistent γH2AX foci appeared to be characteristically enlarged and completely co-localized with 53BP1. The significance of PDDF is still debated: Sedelnikova *et al*. defined them as cryptogenic nuclear foci containing unrepairable DSBs 36, while Rodier *et al*. described them as DNA Segments with Chromatin Alterations Reinforcing Senescence (DNA-SCARES) 42. Moreover, it has been described that changes in chromatin organization are sufficient to induce senescence and are associated with increase in H2AX phosphorylation 43,44. Irrespective of their exact nature, we would like to suggest that PDDF represent another non-canonical role of γH2AX that marks stable chromatin alterations in senescent cells and should be listed among the previously described, novel γH2AX roles 45–47.

Despite SIPS is a potent cell-autonomous tumour suppressor mechanism, the associated SASP, altering the behaviour of neighbouring cells and the quality of tissue environments, exerts cell-non-autonomous effects than can be either beneficial or detrimental 28. In fact, senescent cells with persistent DDR signalling show a robust increase in mRNA levels and protein secretion of numerous cytokines, growth factors and proteases 26,37,48, some of which, like IL6 and IL8, are involved in the reinforcing of the growth arrest 49,50 and exert a tumour promoting role 51. Here, we report that senescence induced by ActD is coupled with transcriptional up-regulation of IL-6 and IL-8, as well as several other chemokines (GRO1, MCP-2, RANTES), inflammatory factors (GM-CSF), metalloproteases (MMP3) and adhesion molecules (ICAM1), confirming the presence of a SASP. The only gene that we did not find up-regulated is SDF1, a growth factor abundantly expressed by hMSCs. Indeed, there are variations in quantity and quality of the SASP that might depend on the cell type and senescence inducer. Albeit SDF1 has been reported to be up-regulated in senescent prostate stromal fibroblasts 52,53, and down-modulated in replicative senescent hMSCs 54, it is not further regulated in ActD-induced senescent hMSCs.

The molecular mechanisms mediating SASP genes up-regulation in hMSCs after the genotoxic stress induced by ActD treatment are not completely elucidated. The most important transcriptional activator of the SASP is the Nuclear factor (NF)-kB (reviewed in 55), whose activation is triggered directly or indirectly by several pathways. In particular, following genotoxic stress ATM has been reported to activate NF-κB signalling *via* post-translational modification of NF-κB essential modulator (NEMO) 56,57. We here observed a very early ATM activation in hMSCs upon ActD treatment and some of the inflammatory cytokines whose mRNA levels were found to be robustly increased (IL-6, IL-8, ICAM1, CXCL1) were described to be ATM-dependent 37. In addition, p53 stabilization/activation was found to induce release of Alarmin HMGB1 that was essential for optimal secretion of IL-6 and MMP3 58 and here we described a markedly increased p53 protein in hMSCs during a 9-day interval from ActD treatment. As for the other up-regulated SASP genes, we can hypothesize the involvement of other, maybe DDR-independent, pathways leading to NF-kB activation, like the one involving p38MAPK that was found to regulate the SASP largely by increasing NF-kB transcriptional activity independently of the DDR 59.

Senescence-associated secretory phenotype has been shown to promote the proliferation of pre-malignant and malignant epithelium 60, enhance invasion 61, induce an epithelial to mesenchymal transition in carcinoma cells 62, increase the growth of xenograft tumours *in vivo* 63 and mediate paracrine transmission of senescence 49. Nevertheless, all these studies were conducted on senescent fibroblasts. On the other hand, it is well documented that also mesenchymal stem cells can play tumour-promoting functions 64–66. Here we found, for the first time to our knowledge, that senescent hMSCs, altering their secretory profile, enhance the migration of two solid tumour derived cell lines: U2OS, an osteosarcoma of mesenchymal origin 67, and CALU-1, a cell line derived from non-small-cell lung tumour known to frequently metastasize to the bone 68. Differently, our *in vitro* analysis highlighted that CM derived from senescent hMSC did not significantly affect their proliferation, at least in the experimental conditions we used. These data suggest that SASP, induced by ActD treatment, increases the complexity of paracrine communication among hMSCs and their physiological/pathological microenvironment, further enhancing their tumour promoting behaviour.

This is the first time that senescence of an adult stem cell is suggested to enhance its tumour-promoting behaviour. It would be interesting to assess whether this feature can be extended to other adult stem cells. A greater understanding of the molecular mechanisms involved in the senescence of hMSCs and careful investigation of the interconnection among senescent and naïve hMSCs secretory phenotypes will provide valuable new insights to comprehend the link with this cancer-promoting behaviour. The impact of chemotherapy treatments on the functional behaviour of hMSCs should be carefully considered with respect to the risk of developing adverse late-occurring chemotherapy complications. Moreover, the predisposition of hMSCs to senescence after genotoxic stress should be carefully evaluated in regenerative approaches that involve *in vitro* cell expansion or manipulation.

## Supporting Information

**Table S1.** List of primers used in qPCR.
