# Supplementary material for: Persistent DNA damage-induced premature senescence alters the functional features of human bone marrow mesenchymal stem cells
Source: J Cell Mol Med. 2015 Jan 26;19(4):734–43. doi: 10.1111/jcmm.12387 (PMC4395188; doi:10.1111/jcmm.12387)
Supplement: Supplementary file 1 [file jcmm0019-0734-sd1.docx]

| Gene Symbol | Gene ID | Forward Primer | Reverse primer |
| --- | --- | --- | --- |
| IL-6 | 3569 | CCAGGAGCCCAGCTATGAACT | CCCAGGGAGAAGGCAACTG |
| IL-8 | 3576 | CTCTTGGCAGCCTTCCTGATT | TTAGCACTCCTTGGCAAAACTG |
| ICAM1 | 3383 | AGCCAGGAGACACTGCAGACA | TGGCTTCGTCAGAATCACGTT |
| GM-CSF | 1437 | CAGTAGAAGTCATCTCAGAAATGTTTGA | GCTCCAGGCGGGTCTGTAG |
| GRO1 | 2919 | CCCACTGCGCCCAAAC | CAGGATTGAGGCAAGCTTTCC |
| RANTES | 6352 | TCTGCGCTCCTGCATCTG | GGGCAATGTAGGCAAAGCA |
| MCP-2 | 6355 | CTCATGGCAGCCACTTTCAG | GCAGGTGATTGGAATGGAAACT |
| SDF1 | 6387 | CACTCCAAACTGTGCCCTTCA | CAATGCACACTTGTCTGTTGTTGT |
| MMP3 | 4314 | TGATGAACAATGGACAAAGGATACA | TTTCATGAGCAGCAACGAGAA |
| Actβ | 60 | GAGTCCGGCCCCTCCAT | GCAACTAAGTCATAGTCCGCCTAGA |

Table 1: List of primers used in qPCR
